# Echocardiography of persistent fifth aortic arch confirmed by computed tomography angiography or surgery in children: a case series

**DOI:** 10.1186/s12872-024-03999-5

**Published:** 2024-06-28

**Authors:** Rui Yuan, Hezhou Li, Juan Wu, Xing Yin

**Affiliations:** 1 Ultrasonic Department, The Third Affiliated Hospital of Zhengzhou University, No. 7, Kangfuqian Street, Erqi District, Zhengzhou, China; 2 Radiology Department, The Third Affiliated Hospital of Zhengzhou University, Zhengzhou, China

**Keywords:** Aortic coarctation, Echocardiography, Pediatrics, Persistent fifth aortic arch

## Abstract

**Background:**

The purpose of this study was to review echocardiography-based diagnosis of persistent fifth aortic arch (PFAA) in children.

**Methods:**

From January 2015 to December 2022, we retrospectively analyzed the echocardiographic findings and the relevant clinical data during follow-up of patients with PFAA who were treated in the Third Affiliated Hospital of Zhengzhou University. The diagnosis was confirmed by computed tomography angiography or surgery.

**Results:**

Seven PFAA cases included two Weinberg type A and five Weinberg type B. The anatomical details of PFAA were assessed using a combination of the long-axis view of the left ventricular outflow tract (from the left high parasternal window) and the long-axis view of the aortic arch (from the suprasternal window). In Weinberg type A, the distal fifth and fourth aortic arches were connected to the descending aorta, which was associated with aortic coarctation. In Weinberg type B, the upper arch of the fourth aorta was interrupted, and only the lower arch of the fifth aorta was connected to the descending aorta. Surgical repair of PFAA was indicated in five patients with blood flow disruption, among which four had good postoperative results and one refused surgery. Two patients with unobstructed PFAA blood flow required follow-up rather than surgery.

**Conclusions:**

It is feasible to diagnose PFAA by echocardiography. Combined application of the high parasternal left ventricular outflow tract view and the suprasternal aortic arch view can improve timely detection of different types of PFAA in children.

## Background

Persistent fifth aortic arch (PFAA) is an unusual malformation of the aortic arch that originates from the ascending aorta opposite or proximal to the innominate artery and terminates in the dorsal aorta or pulmonary artery [1]. PFAA was first reported in humans by R. Van Praagh and S. Van Praagh in 1969 [2]. In PFAA patients, the arch usually forms on the left side and is associated with other cardiac malformations. There are three types of PFAA, as defined by Weinberg [3]. Type A is characterized by a double-lumen aortic arch with both lumina patent. Type B consists of one atretic or interrupted superior arch, and one patent inferior arch. Type C is a systemic-to-pulmonary arterial connection arising proximally to the first brachiocephalic artery. Described as “the great pretender” by Gerlis in 1989 [4], PFAA is often misdiagnosed or missed due to its rarity and a lack of comprehensive understanding. Sometimes PFAA is misdiagnosed as aortic coarctation [5]. At other times, PFAA patients have been regarded as having patent ductus arteriosus because of the similar manifestations [6]. Patients with PFAA usually require surgical intervention during childhood; therefore, early diagnosis and timely treatment are extremely important [7]. In this study, we reviewed echocardiographic manifestations among confirmed PFAA patients.

## Methods

A retrospective analysis was performed on the clinical data of pediatric patients with PFAA who were diagnosed and treated in the Third Affiliated Hospital of Zhengzhou University from January 2015 to December 2022. The diagnosis was based on echocardiographic findings, in combination with findings from other modalities and/or during surgical repair. The study was approved by the Institutional Review Board of the Zhengzhou University Third Hospital. Because the clinical data did not involve names, addresses, or other identifying personal information, and considering the retrospective nature of the investigation, the need for obtaining written consent was waived by the Institutional Review Board of The Third Affiliated Hospital of Zhengzhou University.

Echocardiographic examination was completed on Philips IE33 and EPIQ 7 C ultrasonic diagnostic instruments using an S8-3 probe. The imaging modes included two-dimensional imaging, color Doppler, and spectral Doppler. Pediatric patients were examined in the supine position. Images were taken from subcostal, apical, parasternal, and suprasternal windows. The situs of the atriums, ventricles, great arteries, and the atrioventricular and ventriculo-arterial connections were assessed systemically. The aortic arch and its branches were specifically assessed from two views: (1) from the left high parasternal window: it was in the same plane as the left ventricular outflow tract (LVOT) but focusing on the ascending aorta. We called it the LVOT view; (2) from the suprasternal window: looking at the long axis view of the aortic arch; i.e., the suprasternal view. The stenosis of the aortic arch was evaluated with color Doppler and spectral Doppler.

After PFAA was diagnosed by echocardiography, some of the patients were referred to undergo CT angiography (CTA) to further investigate the anatomical relationship among large blood vessels and their relationship between the heart and surrounding tissues.

## Results

During the study period, echocardiography was performed in approximately 56,800 children to rule out congenital heart disease due to heart murmur, cyanosis, etc. Seven patients with confirmed PFAA were included in the study. No cases of PFAA were missed by preoperative echocardiography. Therefore, the estimated incidence rate of PFAA occurred in approximately 0.01% of the population. Patient characteristics, anatomical diagnoses, and outcomes are summarized in Table 1. All the patients had no family history of congenital heart defects.

**Table 1:** Patient clinical information and echocardiography findings

Case	Gender	Age	Weinberg classification	PFAA position	Associated anomalies	Surgical correction PFAA / Other		Postoperative Hemodynamics / Other	
1	M	5 m	Type A	Left	COA, ASD(II)	Yes, Type A	Yes	Normal	Hyperechogenic masses in the heart
2	M	10 m	Type B	Left	COA	Yes, Type B	No	Normal	-
3	M	27 m	Type B	Left	ASD(II), PDA, VSD	No	Yes	Normal	Complete right bundle branch block
4	F	13 d	Type B	Left	COA, ASD(II)	Yes, Type B	Yes	Normal	-
5	F	4 m	Type B	Right	DORV, PS, VSD	No	Yes	Normal	-
6	M	23 d	Type A	Left	COA, ASD(II)	No	No	Missed visit	-
7	M	1 m	Type B	Left	COA	Yes, Type B	No	Normal	Incomplete right bundle branch block

ASD(II), atrial septal defect secondary hole type; COA, Coarctation of the aorta; d, days; DORV, Double outlet of the right ventricle; F, female; M, male; m, months; PDA, patent ductus arteriosus; PS, pulmonary stenosis; VSD, ventricular septal defect

**Fig. 1:**
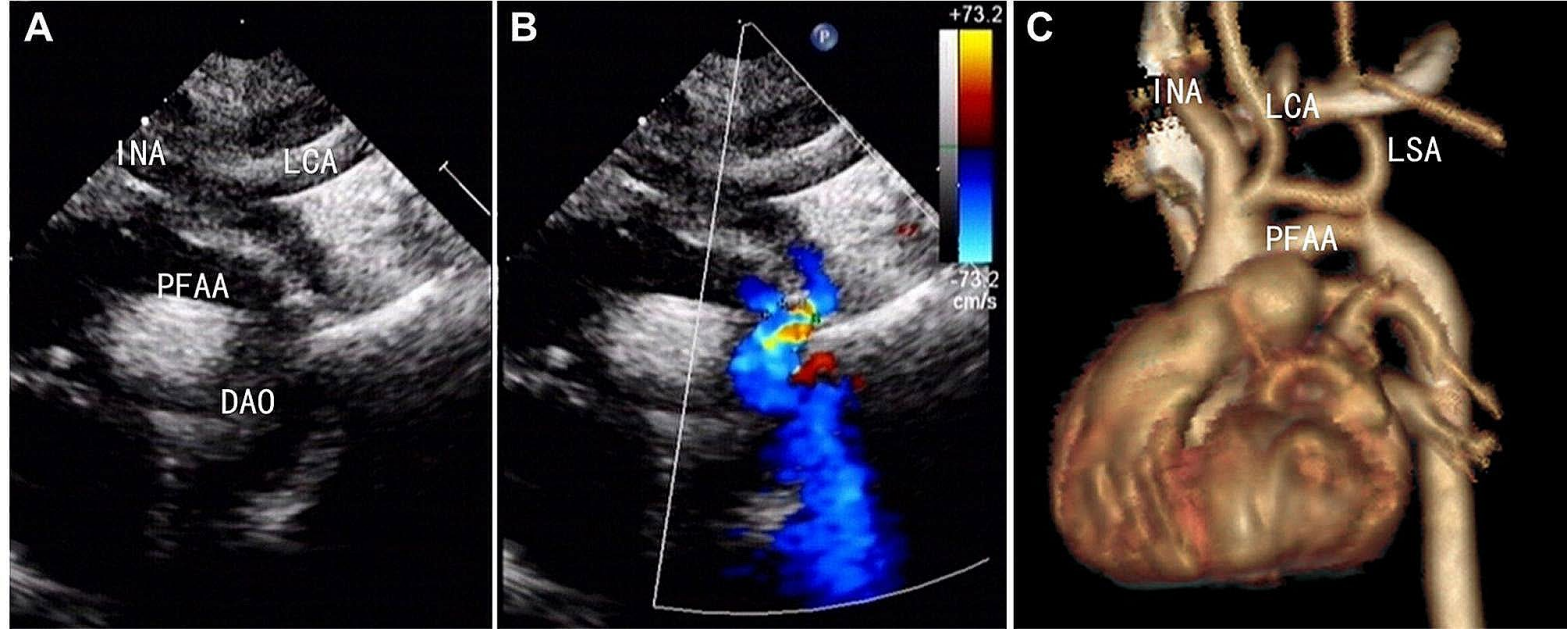
Case 1, Weinberg type A PFAA combined with aortic coarctation. (**A** & **B**): Echocardiography showing the fifth aortic arch originating from the distal ascending aorta and two coexisting and paralleled aortic arches connecting to the descending aorta. **(C)**: Confirmation via computed tomography. DAO: descending aorta; INA, innominate artery; LCA, left common carotid artery; PFAA: persistent fifth aortic arch

**Fig. 2:**
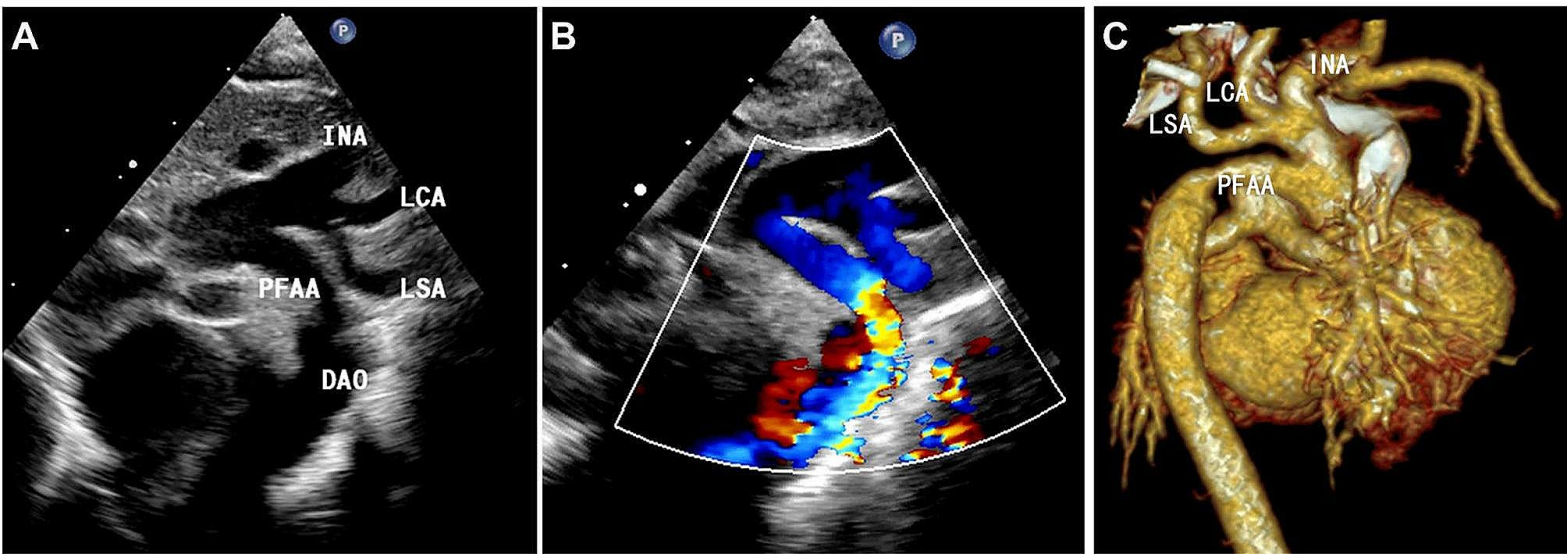
Case 4, Weinberg type B PFAA combined with aortic coarctation. (**A** & **B**): Echocardiography showing the interrupted fourth aortic arch (superior) and fifth aortic arch (inferior) connecting to the descending aorta. **(C)**: Confirmation via computed tomography. DAO, descending aorta; INA, innominate artery; LCA: left common carotid artery; PFAA, persistent fifth aortic arch

In all seven patients, the fifth aortic arch originating from the distal ascending aorta was observed from the LVOT view. In two patients with Weinberg type A, two coexisting and paralleled aortic arches were viewed in the suprasternal view. At the top was the fourth aortic arch, from which the brachiocephalic artery, left common carotid artery, and left subclavian artery stemmed. The fifth aortic arch was inferior to the fourth aortic arch. In both cases, the two arches connected with the descending aorta, where narrowing was confirmed by the small inner diameter of the confluence and turbulent blood flow with color Doppler imaging (Fig. 1). High velocity blood flow of 3.6 m/s and 3.0 m/s, respectively, were revealed in each of the two cases via the Doppler spectrum. In the five patients with Weinberg type B, two paralleled aortic arches were observed from the suprasternal view. However, the superior fourth aortic arch was interrupted distally to the left subclavian artery before giving away the three branches. The fifth aortic arch was inferiorly connected to the descending aorta. Among the five patients with Weinberg type B, three had aortic coarctation (Fig. 2), with high velocity blood flow at 3.4 m/s, 3.6 m/s, and 3.0 m/s, respectively. There was no obstruction of the blood flow in the fifth aortic arch in the other two cases. The diagnosis of PFAA was further confirmed by CTA in six patients and by surgery in four patients.

Five patients with flow obstruction in the fifth aortic arch were indicated for surgical correction. One patient refused surgical treatment, and was lost during the follow-up period. Four patients underwent surgical correction of the fifth aortic arch and its associated cardiac malformations. One patient with Weinberg type A and three with Weinberg type B underwent surgery. Depending on the type of PFAA and the severity of the constriction, the surgeons employed different surgical methods. In some cases, the fifth aortic arch was resected, and the distal end of the descending aorta was raised to the ascending aorta for end-to-side anastomosis. In other cases, the fifth arch was enlarged to form a new aortic arch combined with the ascending aorta. During the postoperative follow-up, no significant residual obstruction or recurrent narrowing of the aortic arch was observed. Two patients did not receive surgical correction of the fifth aortic arch because the blood flow in the fifth aortic arch was unobstructed. In these cases, only associated intracardiac malformations were corrected. No obstruction of the fifth aortic arch occurred during the postoperative follow-up period. One patient was found to have multiple hyperechogenic masses in the interventricular septum and left ventricular posterior wall postoperatively. Two patients developed complete or incomplete right bundle branch block, as observed on electrocardiogram.

## Discussion

During normal embryonic development, a total of six aortic arches form asynchronously, and the arch termed as the bilateral fifth arch eventually disappears [8]. However, in patients with PFAA, the fifth aortic arch fails to degenerate [9]. PFAA is an extrapericardial structure [10, 11]. Hemodynamically, a PFAA can connect the ascending aorta to the descending aorta as a systemic-to-systemic shunt, or it can connect the ascending artery to the left pulmonary artery, functioning as a systemic-to-pulmonary shunt [12]. PFAA can be either left-sided or right-sided but is mostly left-sided in case reports [6, 12–17]. In this study, we reported seven cases of PFAA. In six cases, PFAA was persistent on the left side, and in one case, it was persistent on the right side. Left-sided PFAA occurred in most cases, which was related to the degeneration of the right aortic arch. In all of the patients studied, PFAA connected the ascending aorta to the descending aorta as a systemic-to-systemic shunt.

Weinberg type A PFAA is characterized by the upper and lower segments of the aortic arch forming the commonly known double-lumen aortic arch [18, 19]. The two cases of type A in our study had aortic coarctation. All five cases of Weinberg type B PFAA had interruption of aortic arch (Type A). The LVOT view and suprasternal view are important diagnostic views of PFAA. When the abnormal blood vessel at the distal end of the ascending aorta is first observed from the LVOT view, the location of this vessel, its direction, and how it is connected to other structures should be further evaluated. Differentiation should be made among several abnormalities, including double aortic arch, patent ductus arteriosus, and aortopulmonary septal defect. The suprasternal view is therefore very useful for making such a differentiation. PFAA should be considered if the aortic arch branch vessels originate from the upper aortic arch. Through combined application of color Doppler and spectral Doppler, whether the relevant vessels and their connections had stenosis and the degree of stenosis can be further determined. Computed tomography angiography examination is also recommended, as it is helpful in assessing the development of peripheral blood vessels [13].

Patients with simple PFAA typically have no significant obstruction in the artery and no obvious symptoms, although follow-up is recommended after the initial diagnosis. However, PFAA is often associated with other cardiac or vascular anomalies [20, 21]. In the current study, all cases had combined cardiac anomalies. During the assessment, attention should be paid to exclude combined intracardiac malformations. When fifth aortic dysplasia or other malformations cause hemodynamic abnormalities, surgical correction [11, 22] or stent implantation [23] is required. In the current study, four cases underwent surgical correction of the fifth aortic arch and its associated cardiac malformations. Two cases only received correction of the associated intracardiac malformations because the blood flow of the fifth aortic arch was unobstructed. Postoperative follow-up showed no residual abnormality, except one case had multiple hyperechogenic masses in the heart after surgery, and two cases had abnormal electrocardiograms. The potential etiology of these abnormal findings has not been elucidated, so close monitoring of these patients is needed.

## Conclusions

It is feasible to use echocardiography to diagnose and differentiate among the different types of PFAA. The combined use of the LVOT view and suprasternal view provides a valuable screening approach for the diagnosis of rare diseases like PFAA, which can be further confirmed by CTA. The results of PFAA surgery, which are partially related to the combined malformation, are generally good because of the timely diagnosis. However, long-term follow-up should be conducted to obtain more clinical information.

## Data Availability

The datasets used and/or analysed during the current study are available from the corresponding author on reasonable request.

## Abbreviations

PFAA: Persistent fifth aortic arch
LVOT: Left ventricular outflow tract
